# Unveiling toxigenic *Fusarium* species causing maize ear rot: insights into fumonisin production potential

**DOI:** 10.3389/fpls.2025.1516644

**Published:** 2025-03-21

**Authors:** Harinder Singh, Harleen Kaur, Mandeep Singh Hunjan, Smriti Sharma

**Affiliations:** ^1^ Department of Plant Pathology, Punjab Agricultural University, Ludhiana, Punjab, India; ^2^ Department of Plant Breeding and Genetics, Punjab Agricultural University, Ludhiana, Punjab, India; ^3^ Department of Entomology, Punjab Agricultural University, Ludhiana, Punjab, India

**Keywords:** fumonisins, fum genes, Fusarium ear rot, *Fusarium verticillioides*, maize, quantitative analysis

## Abstract

*Fusarium* species are widespread pathogens of maize (*Zea mays* L.), leading to various diseases throughout the plant’s lifecycle, including Fusarium ear rot (FER), a significant disease that impacts both yield and quality. FER begins at the silking stage when *Fusarium* conidia infect maize silks, particularly in tropical regions where *F. verticillioides* and *F. proliferatum* dominate. These pathogens not only lead to economic losses but also produce mycotoxins such as fumonisins, posing significant health risks to humans and animals. This study aimed to identify toxigenic *Fusarium* species associated with maize ear rot in North India and evaluate their fumonisin production potential under laboratory conditions. Out of the 48 *Fusarium* isolates collected, 40 amplified VERTF-1/2 primers, 41 amplified the *FUM1* gene, while 36 amplified the *FUM13* gene, indicating their potential to produce fumonisins. Sequencing analysis revealed that *F. verticillioides* was the predominant species associated with FER under North Indian conditions, with Fus 48 being identified as *F. proliferatum*. To assess their fumonisin production potential, Fus 15- the most virulent *F. verticillioides* isolate along with *F. proliferatum* isolate- Fus 48 were selected for further analysis. These isolates were artificially inoculated onto maize grains of PMH 1 and PMH 2 hybrids and fumonisin (FB_1_ and FB_2_) levels were quantified using liquid chromatography-mass spectrometry (LC-MS/MS). The results revealed that *F. verticillioides* (Fus 15) exhibited a higher fumonisin production capacity than *F. proliferatum* (Fus 48), with significantly greater fumonisin accumulation in PMH 2 hybrid. This increased fumonisin production in PMH 2 was positively correlated with FER severity under field conditions. Overall, this study provides critical insights into the prevalence and toxigenic potential of *Fusarium* species in North India, which could inform future management strategies to mitigate the impact of FER and its associated mycotoxins on maize production.

## 1 Introduction

*Fusarium*, a ubiquitous pathogen of maize (*Zea mays* L.), consistently associates with the plant throughout its life cycle, inducing various diseases such as seed rot and seedling blight, root rot, post-flowering stalk rots and ear rots (Singh and Kaur, 2023; Kaur et al., 2016; Munkvold, 2003; Logrieco et al., 2002). Among these, Fusarium ear rot (FER) is particularly damaging, significantly reducing both yield and quality of maize. Globally, FER is estimated to cause economic losses of around 26.7%, with potential losses increasing to 48% under favorable environmental conditions (Vigier et al., 2001; Anonymous, 1988). Beyond yield reductions, FER impairs crop quality by producing various mycotoxins which pose serious health risks to humans and animals. Among these, fumonisins are particularly significant contributing to economic losses of up to 46 million US dollars annually (Wu, 2007). First identified by Gelderblom et al. (1988) in South Africa, fumonisins are primarily produced by *Fusarium verticillioides* and *F. proliferatum* under tropical conditions (Huffman et al., 2010; Marasas, 2001). Their presence raises major health concerns due to potential links with human cancer, neural tube defects and other birth abnormalities, and its toxicity to domestic livestock (Missmer et al., 2006; Logrieco et al., 2003; Gelderblom et al., 1988). In India, an outbreak of fumonisin toxicosis affecting 10,000 hens was reported in Andhra Pradesh in the last quarter of 1995 (Kumar et al., 1997). To mitigate these losses, it is crucial to detect fumonisins using molecular tools. Fumonisin production is regulated by the “*FUM* cluster”, a set of 16 co-regulated genes essential for biosynthesis, including the key *FUM1* gene, which encodes polyketide synthase. The utilization of molecular markers for identifying toxigenic strains and detecting the *FUM* genes across species, represents the most effective strategy for elucidating their mycotoxin potential (Patiño et al., 2004; Proctor et al., 1999; Stepien et al., 2011).

The production of fumonisins in standing maize crop is closely linked with FER severity. This disease typically initiates at the silking stage, as airborne conidia of the fungus land on the silks and enter the cob through insect-caused injuries or direct contact with the silks. In addition, systemic infection can occur via contaminated seeds, which leads to early establishment of the pathogen before the silking stage (Foroud et al., 2014). Various *Fusarium* spp. *viz., F. subglutinans, F. graminearum, F. cerealis, F. avenaceum* and *F. equiseti* have been found associated with ear rot throughout the world. However, under tropical environmental conditions, two *Fusarium* spp. *viz., F. verticillioides* and *F. proliferatum* belonging to *Fusarium* section Lesiola are more predominant (Pfordt et al., 2020). Despite the global significance of these pathogens, knowledge about the prevalence of toxigenic *Fusarium* species associated with maize ear rot in North India remains limited. This study aimed to bridge this gap by identifying *Fusarium* species using species-specific and gene-specific primers and evaluating their fumonisin production potential under laboratory conditions. The findings aim to enhance our understanding of the current status of toxigenic *Fusarium* species and guide future management strategies.

## 2 Materials and methods

### 2.1 Collection, isolation and maintenance of *Fusarium* isolates

The cob samples showing peculiar symptoms of ear rot were collected from major maize growing areas of Punjab state *viz.*, Gurdaspur, Hoshiarpur, Shaheed Bhagat Singh Nagar, Rupnagar, Jalandhar, Kapurthala, Ludhiana during Spring and *Kharif* seasons of 2020 as well as from Sirmour district of Himachal Pradesh during *Kharif* 2021. The isolations were performed on potato dextrose agar (PDA) medium, resulting in the recovery of 48 *Fusarium* isolates (Fus 1 to Fus 48). The pure cultured isolates were maintained at 4°C for further studies.

### 2.2 Isolation of fungal genomic DNA

For extraction of fungal genomic DNA, the pure cultures of all 48 *Fusarium* isolates were grown on 100 ml Potato Dextrose Broth (PDB) medium and were incubated at 25 ± 2°C for 10 days. Ten days old fungal mycelial mat obtained through filtering on Whatman filter paper No. 1 was ground to fine powder using liquid nitrogen and transferred into 2 ml eppendorf tube containing 800 µl preheated (65°C) Cetyl trimethyl ammonium bromide (CTAB) extraction buffer and mixed thoroughly with buffer. Fungal DNA from all the isolates was isolated using CTAB method as described by Saghai-Maroof et al. (1984). The extracted DNA was subjected to RNAse (2 µl) treatment and the samples were incubated at 37°C for 45 minutes. Quantity and quality of DNA was checked on Eppendorf Bio Spectrometer and 0.8% agarose gel. The DNA samples were then stored at -20°C for further studies.

### 2.3 Identification of fumonisin producing isolates of *Fusarium* spp.

Using VERTF*-*1/2primers: The fumonisin producing *F. verticillioides* isolates were distinguished from non-producers using a set of VERTF-1/2 primers (F- GCGGGAATTCAAAAGTGGCC, R- GAGGGCGCGAAACGGATCGG) as described by Patiño et al. (2004). Polymerase chain reaction (PCR) amplification was carried out in an Eppendorf Mastercycler Pro S (Eppendorf, Germany) using the same amplification profile as stated by Patiño et al. (2004). PCR analysis was carried out in the reaction volume of 25 µl/sample containing-0.4 µl of GoTaq™ DNA polymerase (Promega Inc.), 5 µl of 1X GoTaq™ Reaction Buffer (Green), 1.5 µl of 2.5 mM MgCl_2_ (Promega Inc.), 1.5 µl each of 10 pmol forward/reverse primers, 0.5 µl of 0.1 mM dNTPs (Promega Inc.), 2 µl of template DNA and 12.60 µl of nuclease free water (Promega Inc.).

Using *FUM* gene specific primers: The ability of *Fusarium* isolates to produce fumonisins was studied by amplification and sequencing of segments of two *FUM* genes namely- *FUM1* and *FUM13.* Both these genes were amplified using sets of FUM1F1/R2 (F-CACATCTGTGGGCGATCC, R-ATATGGCCCCAGCTGCATA) (Stepien et al., 2011), and FUM13F/13R (F-AGTCGGGTCAAGAGCTTGT, R-TGCTGAGCCGACATCATAATC) (Ramana et al., 2011), respectively. *In vitro* amplification was performed in an Eppendrof master cycler Pro S (Eppendorf, Germany) using *FUM1* and *FUM13* gene specific primers. PCR reaction mixture consisted of 25 µl volume/sample as mentioned above. The amplification of *FUM1* gene was performed with one cycle of initial denaturation at 94°C for 5 minutes followed by 35 cycles of denaturation at 94°C for 45 seconds, annealing at 55°C for 45 seconds, extension at 72°C for 2 minutes with one cycle of final extension at 72°C for 7 minutes. The *FUM13* gene was amplified using same profile as stated by Ramana et al. (2011).

The amplified PCR products were visualized on 1% agarose gel under UV light and photographed using SYNGENE gel documentation system with “GeneSnap” software program. The band size of PCR products was determined by comparing with known marker (100 bp ladder, G Biosciences, USA).

### 2.4 Sequencing of isolates

Four representative isolates (Fus 15, Fus 28, Fus 44 and Fus 45) showing positive amplification for both *FUM1* and *FUM13* genes along with Fus 48 isolate amplifying *FUM1* gene were selected for sequencing. The amplified PCR products were purified by using NucleoSpin Gel and PCR Clean-up kit (Macherey-Nagel, Germany), following the manufacturer’s instructions. Around 18 µl of positive purified products (100ng/µl) of these five isolates were sequenced (Barcode Biosciences Bangalore). The retrieved forward and reverse sequences were aligned using BioEdit software, and saved as a single contig file. Contigs were generated and screened against online Gene DNA sequence database using BLASTn (Nucleotide Basic Local Alignment Search Tool) program available at NCBI website (https://blast.ncbi.nlm.nih.gov). Matching sequences were retrieved from BLASTn analysis and further used for phylogenetic analysis or sequence comparison.

**Statistical analysis:** Multiple sequence alignment (MSA) was performed using Mega X (Kumar et al., 2018) with Neighbor- Joining Tree option. The phylogenetic trees were constructed and the sequences of the identified *Fusarium* species were submitted to NCBI nucleotide database.

### 2.5 Quantitative analysis of fumonisins (FB_1_ and FB_2_) produced in artificially inoculated maize grains

*Selection of maize hybrids*: Two maize hybrids- PMH 1 and PMH 2 differing in maturity groups as well as response to *Fusarium* spp. were selected for the quantitative analysis of fumonisins. PMH 1, late-maturity hybrid known for its resistance to *F*. *verticillioides* (Kaur et al., 2016), and PMH 2, early-duration hybrid susceptible to FER (Singh and Kaur, 2024), were selected to understand the relationship between FER infection and fumonisin accumulation.

*Selection of Fusarium isolates*: Among *F. verticillioides* isolates, the most virulent isolate- Fus 15 and *F. proliferatum* isolate- Fus 48 showing higher FER severity under artificially inoculated field conditions were selected for quantitative analysis of fumonisins.

*Estimation of fumonisins*: The fumonisin content of FB_1_ and FB_2_ in artificially inoculated maize grains of PMH 1 and PMH 2 was analyzed by using triple quadrupole liquid chromatography mass spectrometer (LC MS MS) on LCMS-MS-8045 (Shimadzu). The control treatment consisted of seed mixed with sterile distilled water. These inoculated grains were incubated at 25 ± 2°C and sampling for quantification of fumonisin production was done at 7, 14, 21 and 28 days after inoculations. Two dilutions of standards FB_1_ and FB_2_ (1 ppm and 0.1 ppm) (LGC Standards GmbH, Germany) were prepared with HPLC grade acetonitrile from stock solution for constructing a calibration curve. The sample preparation for fumonisins estimation was done as stated by Li et al. (2012). Recovery experiments were carried out to know the efficacy of the analytical method used and samples (5 g) of healthy maize grains were fortified with fumonisin B_1_ and B_2_ at level of 10, 50 and 100 µg kg^-1^ with three replications of each. The sample was processed and per cent recovery was calculated. The chromatographic separation of analytes was done using C_18_ (2.1 ×150 mm) 2μm (HSS) column, in positive ion mode using multiple reaction monitoring (MRM) of the transitions m/z (**Table 1**). The mobile phase of 10 mM ammonium formate+0.1% formic acid in water: 0.1% formic acid in methanol (A: B 10: 90 v/v) was used with flow rate of 250 µL/min. MS conditions were: column temperature 40°C, interface temperature 250°C, desolvation temperature 444°C, DL temperature 250°C, heat block temperature 300°C, nebulizing gas flow rate at 2.5 L/min, heating gas flow rate at 5 L/min and drying gas flow rate at 5 L/min. The precursor ions m/z and collision energy for each of the compound is given in **Table 1**. Lab solutions software was used to acquire and analyze the data. The fumonisins content were estimated by comparing peak height/peak area of the standard with that of unknown or spiked samples ran under identical conditions. The data was analyzed for mean ± standard error for both fumonisins-FB_1_ and FB_2_.

**Table 1:** The precursor ions and collision energy for FB_1_ and FB_2_ estimation.

Fumonisins	Precursor	Product	Q1	CE	Q3
FB_1_	722.40	352.40	-20	-38	-17
	722.40	334.40	-34	-44	-23
FB_2_	706.10	336.20	-32	-40	-23
	706.10	318.30	-24	-41	-21


$$\text{Residue level }\left(\text{mg}/\text{kg}\right)=\frac{\text{Area of sample}}{\text{Area of standard}}\times \frac{\text{ng of standard injected}}{\text{µl of sample injeted}}\times \frac{\text{Final volume taken}}{\text{Total weight}}$$

## 3 Results

### 3.1 Identification of fumonisin producing *Fusarium* isolates

**Figure 1:**
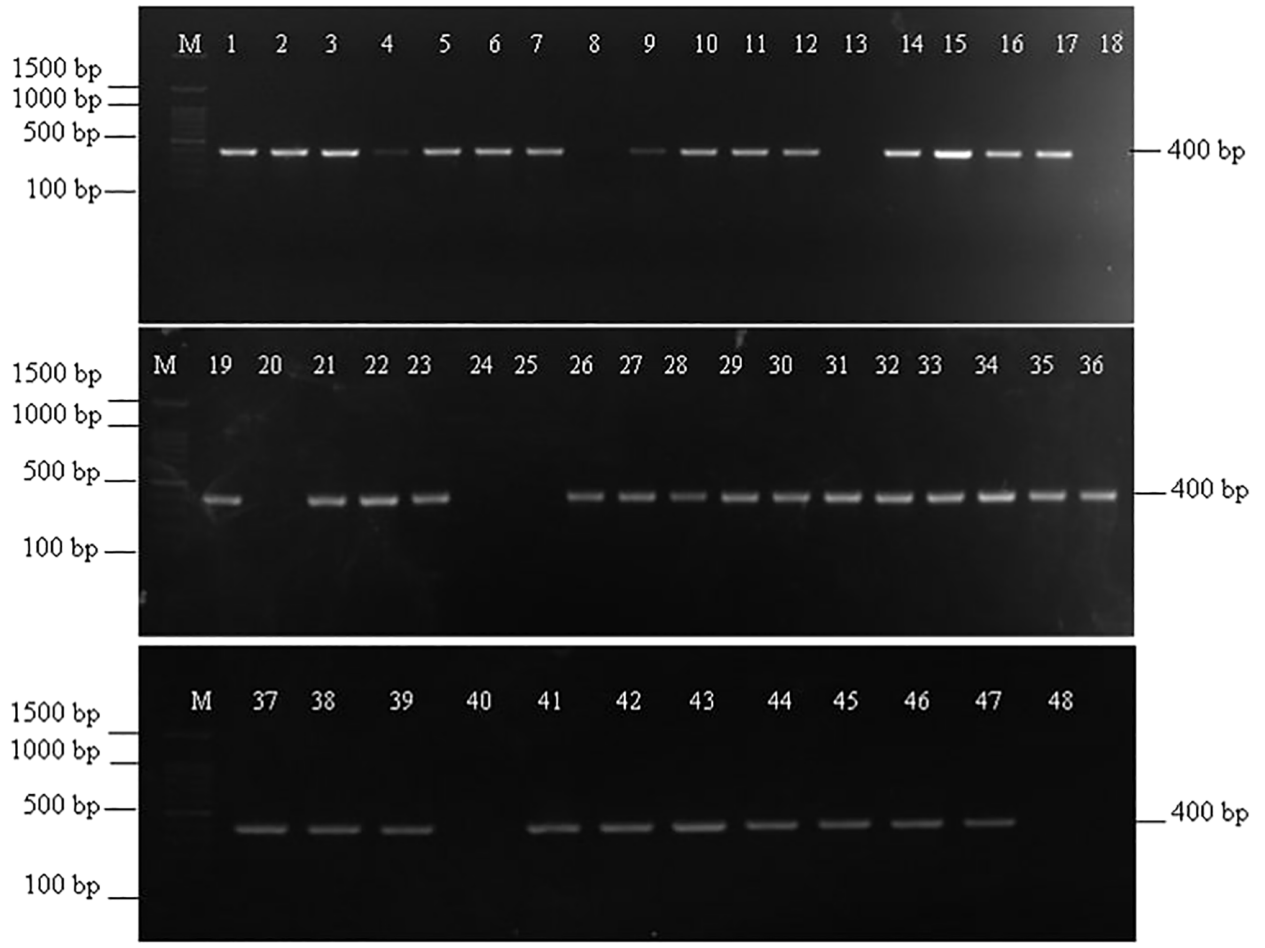
Amplification of fumonisin producing *F. verticillioides* isolates using VERTF-1/2 primers showing amplification band of 400 bp, Lane M contains- 100 bp ladder; Lane 1-48 contains DNA from *Fusarium* isolates Fus 1 to Fus 48 from different locations.

**Figure 2:**
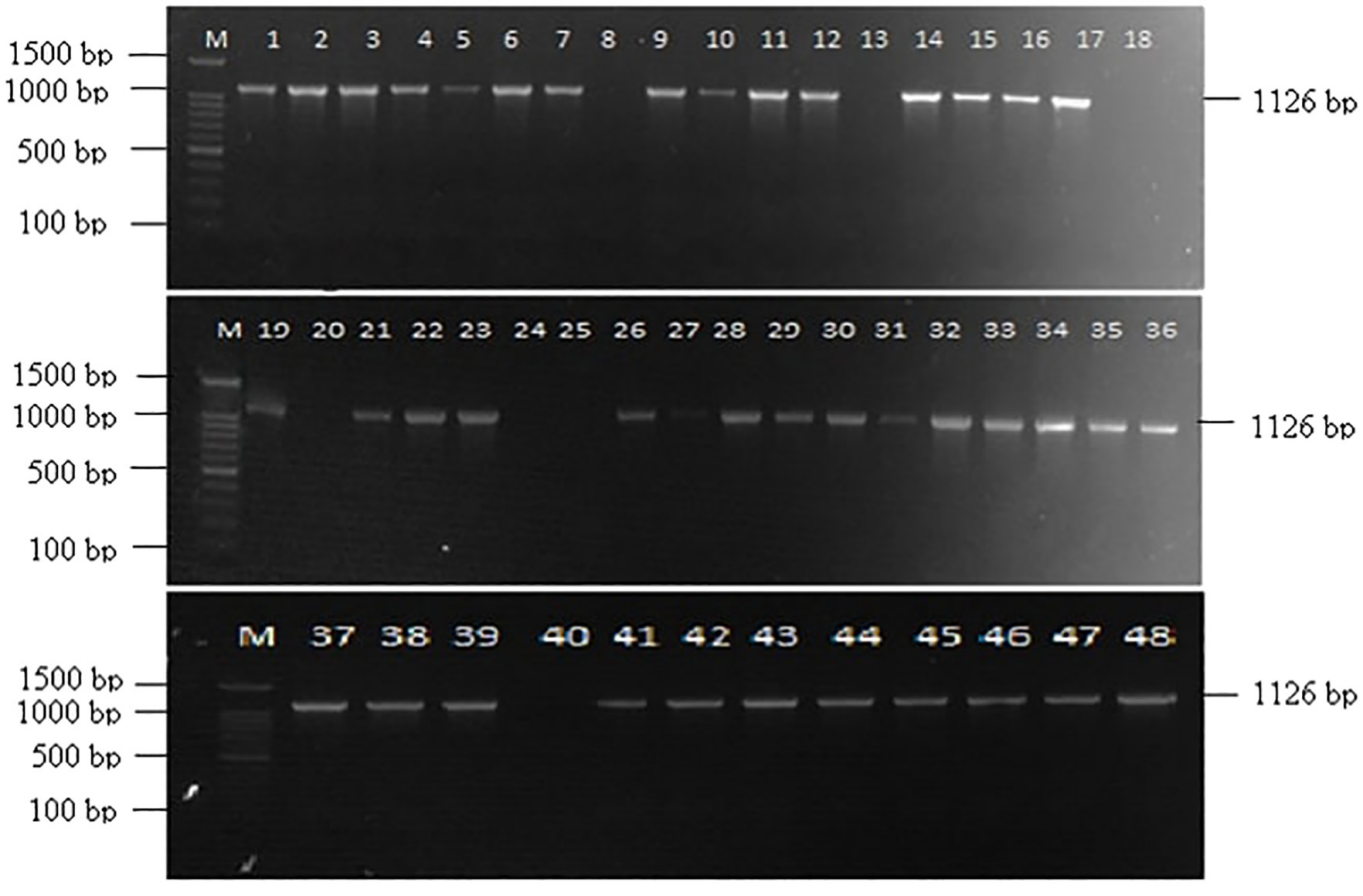
Amplification of fumonisin producing *Fusarium* isolates using *FUM1* gene specific primers showing amplification band of 1126 bp, Lane M contains- 100 bp ladder; Lane 1-48 contains DNA from *Fusarium* isolates Fus 1 to Fus 48 from different locations.

**Figure 3:**
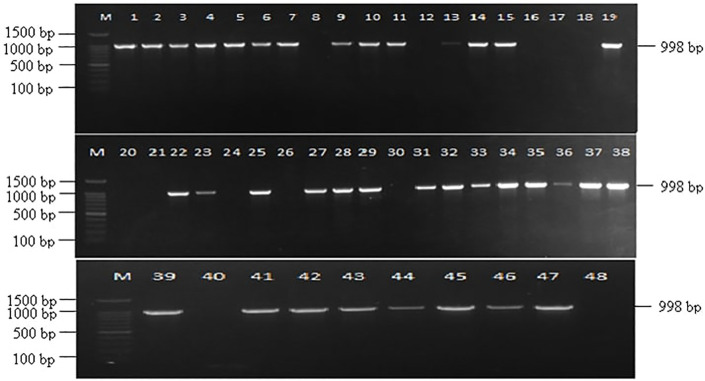
Amplification of fumonisin producing *Fusarium* isolates using *FUM13* gene specific primers showing amplification band of 998 bp, Lane M contains- 100 bp ladder; Lane 1-48 contains DNA from *Fusarium* isolates Fus 1 to Fus 48 from different locations.

Out of 48 *Fusarium* isolates collected from maize ear rot samples, 40 showed a successful amplification with VERTF-1/2 primers, producing a 400 bp band, confirming them as fumonisin-producing *F. verticillioides* strains (**Figure 1**). Additionally, *FUM1* gene specific primers amplified a band of 1126 bp in 41 isolates (**Figure 2**). Moreover, *FUM13* specific primer produced a band of 998 bp in 36 *Fusarium* isolates, and the rest of 12 isolates lacking this gene did not show any amplification (**Figure 3**). Data in **Table 2** showed that five isolates (Fus 8, Fus 18, Fus 20, Fus 24 and Fus 40) did not show any amplification with VERTF-1/2 primers, *FUM1* and *FUM13* genes. Only six isolates *viz*., Fus 12, Fus 16, Fus 17, Fus 21, Fus 26 and Fus 30 showed amplification with VERTF-1/2 primers and *FUM1* gene and only two isolates *viz.* Fus 13 and Fus 25 gave amplification with FUM13F/13R primers. One *F. proliferatum* isolate -Fus 48 showed amplification only with FUM1F1/R2 primer; there was no amplification with VERTF-1/VERTF-2 and FUM13F/13R primers (**Table 2**).

**Table 2:** Grouping of *Fusarium* isolates based on *FUM1* and *FUM13* genes.

*Fusarium* species	Isolates	VERTF-1/2 primers	*FUM1* gene	*FUM13* gene	Frequency of isolates (%)
*F. verticillioides*	Fus 1, Fus 2, Fus 3, Fus 4, Fus 5, Fus 6, Fus 7, Fus 9, Fus 10, Fus 11, Fus 14, Fus 15, Fus 19, Fus 22, Fus 23, Fus 27, Fus 28, Fus 29, Fus 31, Fus 32, Fus 33, Fus 34, Fus 35, Fus 36, Fus 37, Fus 38, Fus 39, Fus 41, Fus 42, Fus 43, Fus 44, Fus 45, Fus 46, Fus 47	+	+	+	70.83
	Fus 8, Fus 18, Fus 20, Fus 24, Fus 40	-	-	-	10.41
	Fus 12, Fus 16, Fus 17, Fus 21, Fus 26, Fus 30	+	+	-	12.50
	Fus 13, Fus 25	-	-	+	4.16
*F. proliferatum*	Fus 48	-	+	-	2.08

+ Present, - Absent.

### 3.2 Sequencing and confirmation of *Fusarium* spp.

The sequences of *FUM1* gene for Fus 15, Fus 28, Fus 44 and Fus 45 submitted to BLAST revealed 99-100% similarity with *F. verticillioides* (having reference id XM_018886754.1). However, Fus 48 had shown 98% similarity with *F. proliferatum* (reference id CP128308.1) when subjected to BLAST. The *FUM13* gene sequences of Fus 15, Fus 28, Fus 44 and Fus 45 isolates showed 99-100% similarity with *F. verticillioides* (reference id XM_018886761.1). The phylogenetic analysis of *Fusarium* isolates for both *FUM1* (5 isolates) and *FUM13* (4 isolates) genes resulted in 99-100% similarity with respective species (**Supplementary Figures S4**, **S5**). The above studies confirmed that *FUM1* and *FUM13* genes were present in 85.41% and 75% of the isolates, respectively. Since these genes are essential for fumonisin biosynthesis, their presence suggests a potential for toxin production. Furthermore, to assess and confirm the mycotoxin production potential of these isolates fumonisin content was analyzed using LC MS MS method.

### 3.3 Quantitative analysis of fumonisin content

**Figure 4:**
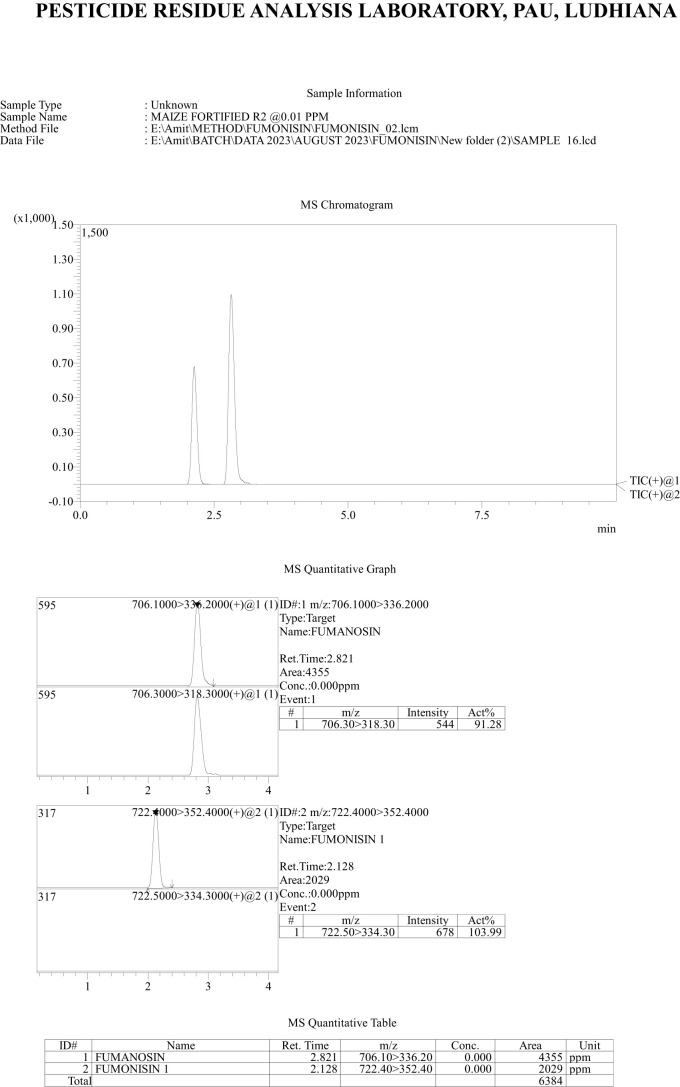
Chromatogram showing response of fumonisins (FB_1_ and FB_2_) standards at 10 µg kg-1.

**Figure 5:**
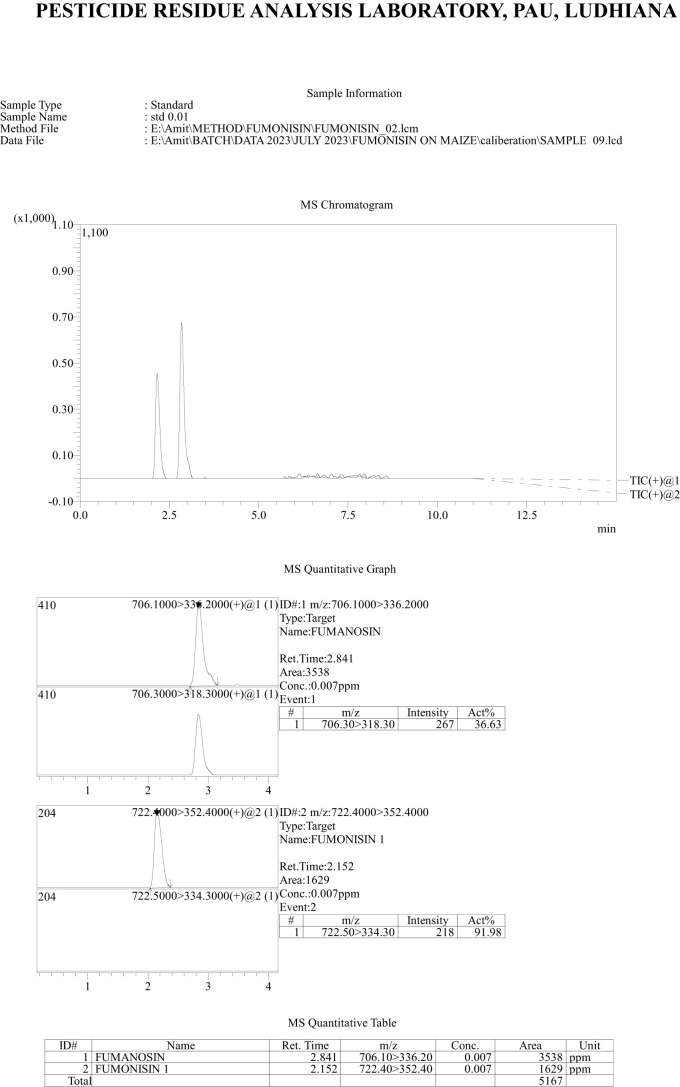
Chromatogram of recovery of fumonisins- FB_1_ and FB_2_ at 10 µg kg^-1^ level of fortification.

**Figure 6:**
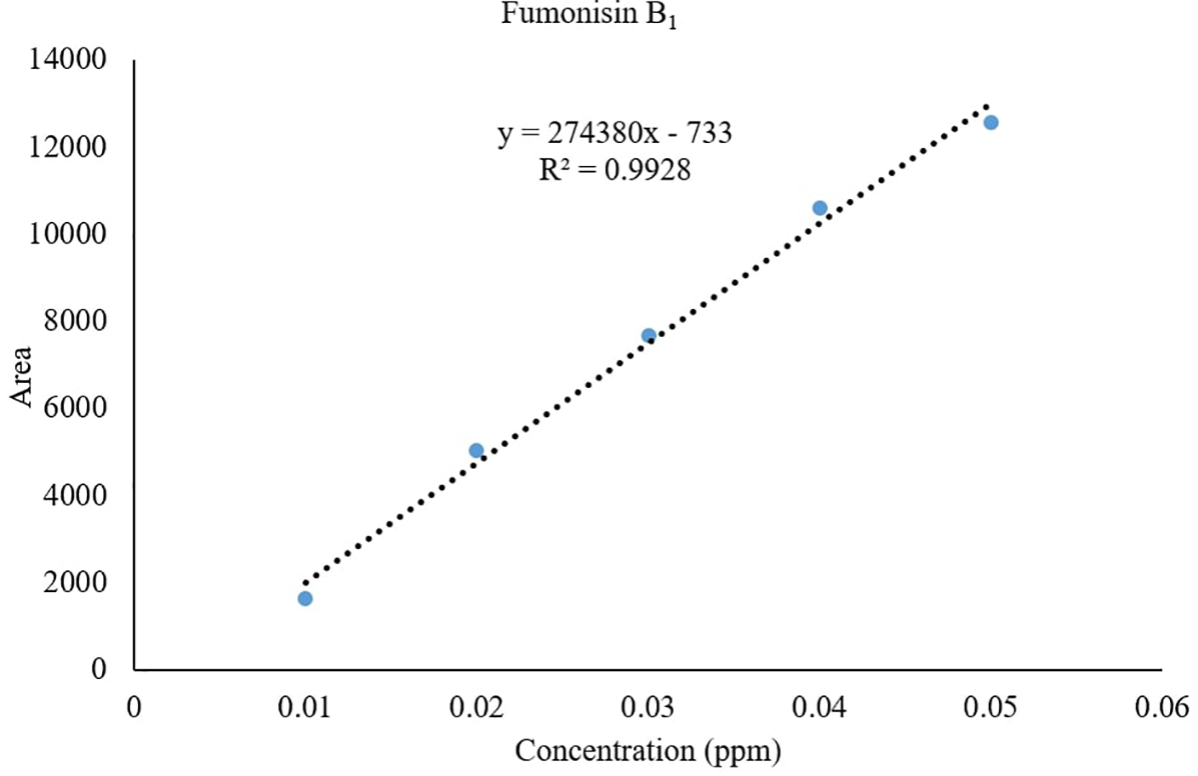
Linearity of FB_1_ standard.

**Figure 7:**
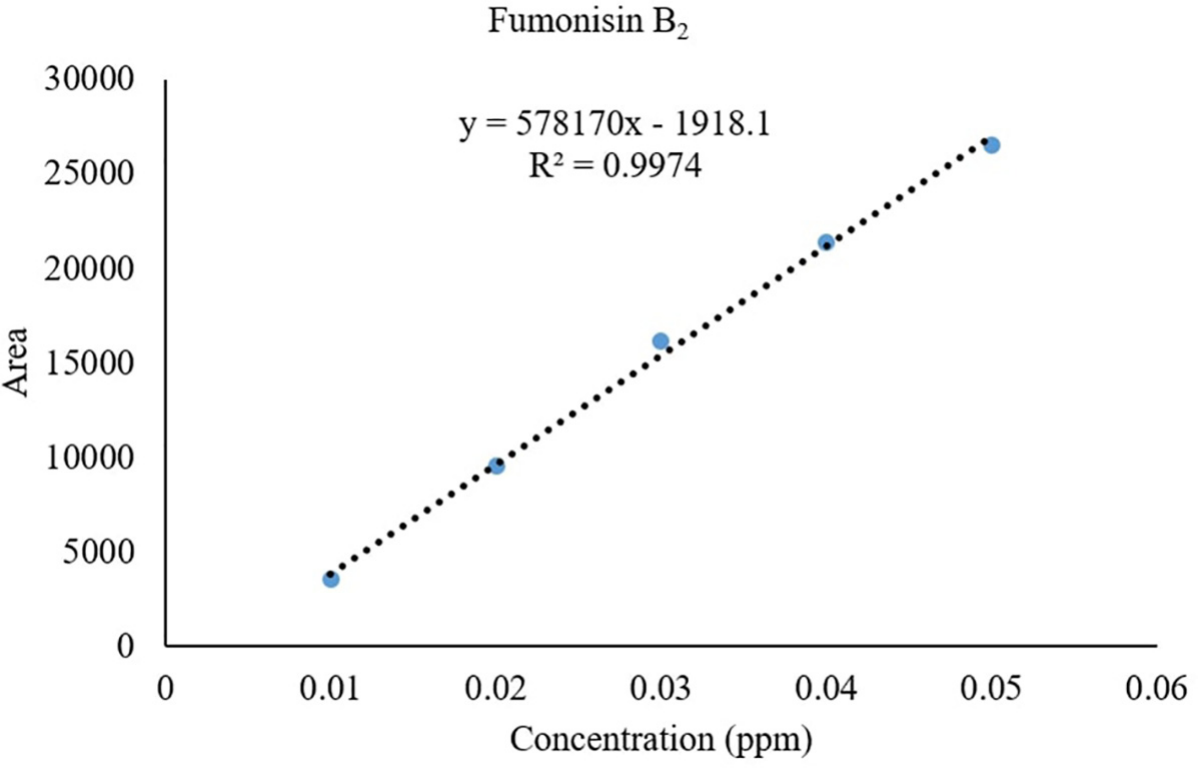
Linearity of FB_2_ standard.

*Recovery of fumonisins*: The average recoveries of FB_1_ from maize grains fortified at 10, 50, and 100 µg/kg were 75.67%, 90.63%, and 85.14%, respectively. For FB_2_, the recoveries were 73.01%, 98.40%, and 82.04% at the same fortification levels (**Table 3**). The control samples from untreated grains and reagent blanks were also processed in the same way so as to find out the interferences, if any, due to the substrate and reagents, respectively and no interferences were observed at the retention time (RT) of FB_1_ and FB_2_ (**Figure 4**). The recoveries obtained were within the international acceptable levels. The limit of quantitation (LOQ) of the method was 10 µg kg^-1^ (**Figure 5**). The linearity curves for both the fumonisins standards (FB_1_ and FB_2_) were constructed by injecting 10, 20, 30, 40 and 50 ng/ml of each of the standard and the response was found to be linear with R^2^ values of 0.9935 and 0.9951 for FB_1_ and FB_2_, respectively (**Figures 6**, **7**).

**Table 3:** Recovery per cent of FB_1_ and FB_2_ in maize grains at different fortification levels.

Level of fortification (µg kg^-1^)	Fumonisin B_1_ (FB_1_)		Fumonisin B_2_ (FB_2_)	
	Recovery (%)	Mean ± SE	Recovery (%)	Mean ± SE
10	74.73	75.67 ± 1.03	73.85	73.01 ± 1.83
	76.78		70.90	
	75.50		74.27	
50	90.62	90.63 ± 0.30	97.54	98.40 ± 1.45
	90.32		97.60	
	90.95		100.08	
100	85.45	85.14 ± 0.47	82.94	82.04 ± 2.30
	84.60		83.76	
	85.38		79.42	

*Fumonisin content in maize samples*: The data presented in **Table 4** and **Table 5** revealed that FB_1_ and FB_2_ were produced by both the isolates of *Fusarium* species. When comparing the isolates, *F. verticillioides* isolate (Fus 15) produced greater quantity of fumonisins than *F. proliferatum* isolate (Fus 48) across all four sampling intervals. Seven days after inoculation, Fus 15 isolate produced lower concentration of FB_1_ in the PMH 1 (0.03 mg kg^-1^) and PMH 2 (1.06 mg kg^-1^) hybrids. By 21 days after inoculation, FB_1_ levels rose to 17.29 mg kg^-1^ in PMH 1 and 96.93 mg kg^-1^ in PMH 2 (**Table 4**) (**Supplementary Figure S6**). A similar trend was observed with Fus 48 isolate. The perusal of data presented in **Table 5** revealed that at seven days after inoculation, Fus 15 isolate produced FB_2_ only in PMH 2 grains (0.22 mg kg^-1^), however its quantity in PMH 1 hybrid was below LOQ. Upto 14 days after inoculation, FB_2_ production by Fus 48 isolate was below LOQ in both hybrids.

**Table 4:** Fumonisin FB_1_ (mg kg^-1^) production in maize grains artificial inoculated with *Fusarium* isolates.

Days after inoculation	Fumonisin FB_1_ (mg kg^-1^) in PMH 1 grains		Fumonisin FB_1_ (mg kg^-1^) in PMH 2 grains	
	*Fusarium verticillioides* (Fus 15)	*Fusarium proliferatum* (Fus 48)	*Fusarium verticillioides* (Fus 15)	*Fusarium proliferatum* (Fus 48)
7	0.04	0.013	1.092	0.038
	0.03	0.012	1.101	0.044
	0.04	0.012	0.994	0.034
Mean ± SE	0.03 ± 0.003	0.01 ± 0.000	1.06 ± 0.03	0.04 ± 0.002
14	1.451	0.012	5.694	0.071
	1.231	0.01	5.871	0.064
	1.217	0.013	4.308	0.07
Mean ± SE	1.29± 0.07	0.01 ± 0.000	5.29 ± 0.49	0.06 ± 0.002
21	16.674	0.341	95.481	0.561
	17.698	0.343	97.88	0.458
	17.514	0.335	97.449	0.529
Mean ± SE	17.29 ± 0.31	0.33 ± 0.002	96.93 ± 0.73	0.51 ± 0.03
28	25.402	0.716	Rotting of kernels	1.393
	20.179	1.045		1.399
	26.518	1.019		1.385
Mean ± SE	24.03 ± 1.92	0.92 ± 0.10		1.39 ± 0.004

Limit of quantitation (LOQ)=10 µg kg^-1^.

**Table 5:** Fumonisin FB_2_ (mg kg^-1^) production in maize grains artificial inoculated with *Fusarium* isolates.

Days after inoculation	Fumonisin FB_2_ (mg kg^-1^) in PMH 1 grains		Fumonisin FB_2_ (mg kg^-1^) in PMH 2 grains	
	*Fusarium verticillioides* (Fus 15)	*Fusarium proliferatum* (Fus 48)	*Fusarium verticillioides* (Fus 15)	*Fusarium proliferatum* (Fus 48)
7	<LOQ	<LOQ	0.237	<LOQ
	<LOQ	<LOQ	0.221	<LOQ
	<LOQ	<LOQ	0.251	<LOQ
Mean ± SE	<LOQ	<LOQ	0.221 ± 0.008	<LOQ
14	0.369	<LOQ	1.356	<LOQ
	0.408	<LOQ	2.009	<LOQ
	0.41	<LOQ	1.215	<LOQ
Mean ± SE	0.395 ± 0.01	<LOQ	1.52 ± 0.24	<LOQ
21	5.433	0.056	24.324	0.081
	5.553	0.055	22.344	0.065
	5.318	0.053	21.188	0.078
Mean ± SE	5.43 ± 0.06	0.05 ± 0.000	22.61 ± 0.91	0.07 ± 0.004
28	10.753	0.066	Rotting of kernels	0.082
	8.29	0.052		0.076
	10.987	0.026		0.075
Mean ± SE	10.01 ± 0.86	0.04 ± 0.01		0.07 ± 0.002

Limit of quantitation (LOQ)= 10 µg kg^-1^.

PMH 2 consistently exhibited higher levels of fumonisins (FB_1_ and FB_2_) than PMH 1 across all sampling days. In both hybrids, fumonisin levels gradually increased with inoculation time, reaching their peak at 21 days, with PMH 2 showing the highest FB_1_ (96.93 mg/kg) and FB_2_ (22.61 mg/kg) accumulation.

## 4 Discussion

The study explored the fumonisin production potential of *Fusarium* species, particularly focusing on *F. verticillioides* and *F. proliferatum*. The results demonstrated that the majority of *F. verticillioides* isolates (85.10%) were fumonisin producers, as confirmed by VERTF-1/2 primers. These findings align with previous studies, such as those by Magculia and Cumagun (2011), who found that 38 out of 49 F*. verticillioides* isolates were potential fumonisin producers based on the amplification of a 400 bp DNA fragment specific to these primers. Similarly, Maheshwar et al. (2009) and Szecsi et al. (2011) identified fumonisin production in F*. verticillioides* using species-specific primers, further corroborating the utility of VERTF-1/2 in detecting toxigenic strains.

The fumonisin biosynthesis pathway, driven by the *FUM* gene cluster, plays a crucial role in determining the toxigenic potential of *Fusarium* species (Proctor et al., 2003; Sánchez-Rangel et al., 2005; Niehaus et al., 2017; Choi et al., 2018; Zyl et al., 2019; Mohiddin et al., 2021). The study’s focus on *FUM1* and *FUM13* genes, which are vital for fumonisin production, is consistent with findings from previous research (Qiu et al., 2020; Kaur, 2015; Proctor et al., 2013; Medina et al., 2013; Ramana et al., 2011). Notably, the presence of these genes was also detected in other *Fusarium* species within the FFSC, such as *F. proliferatum* and *F. fujikuroi*, as reported by Choi et al. (2018). However, it is important to note that the detection of the *FUM* gene cluster does not always correlate with fumonisin production, as some non-detectable fumonisin-producing strains also harbor these genes (Sultana et al., 2019). Variations in fumonisin production among *Fusarium* species and even among different isolates of the same species have been attributed to factors such as pathogen strain differences, environmental conditions, varietal susceptibility and the origin of the species (Singh and Kaur, 2024; Kamle et al., 2019). In this study, *F. verticillioides* exhibited a higher fumonisin production potential than *F. proliferatum*, which is consistent with previous studies (Ismail et al., 2017; Ghiasian et al., 2005; Tancic et al., 2012; Acuna et al., 2005; Logrieco et al., 2002; Covarelli et al., 2012). The study also observed that fumonisin B_1_ production increased with inoculation time, a trend that has been documented in several studies (Altinok, 2009; Dilkin et al., 2002; Bailly et al., 2002).

Resistance to FER is polygenic in nature and no maize cultivar has been reported to exhibit complete resistance. Furthermore, no information is available regarding FER resistance in maize under North Indian conditions. Based on our previous studies in north zone (Kaur et al., 2016; Singh and Kaur, 2024) hybrids showed varied response against *F. verticillioides* infection. Two hybrids- PMH 1 exhibiting resistance while PMH 2 showing susceptibility to *F. verticillioides* were further selected for fumonisin analysis. The PMH 1 hybrid exhibited lower fumonisin accumulation compared to the PMH 2 hybrid, suggesting a potential resistance mechanism against fumonisin production in PMH 1. This finding aligns with previous reports that have shown a positive correlation between FER severity and fumonisin concentration (Junior et al., 2019; Netshifhefhe et al., 2018; Maschietto et al., 2017; Balconi et al., 2014; Afolabi et al., 2007).

## 5 Conclusion

The study reinforces the role of molecular techniques, particularly the use of species-specific primers and the analysis of the *FUM* gene cluster, in differentiating between toxigenic and non-toxigenic *Fusarium* strains. The results also underscore the variability in fumonisin production among *Fusarium* species and isolates, influenced by genetic and environmental factors. The observed correlation between FER severity and fumonisin concentration highlights the importance of developing resistant maize hybrids to mitigate the risks associated with fumonisin contamination.

## Data Availability

The datasets presented in this study can be found in online repositories. The names of the repository/repositories and accession number(s) can be found in the article/**Supplementary Material**.
