# Supplementary material for: Unveiling toxigenic Fusarium species causing maize ear rot: insights into fumonisin production potential
Source: Front Plant Sci. 2025 Mar 21;16:1516644. doi: 10.3389/fpls.2025.1516644 (PMC11968736; doi:10.3389/fpls.2025.1516644)
Supplement: Supplementary file 2 [file DataSheet2.pdf]

PESTICIDE RESIDUE ANALYSIS LABORATORY, PAU, LUDHIANA

Sample Information

Sample Type	: Standard
Sample Name	: std 0.01
Method File	: E:\Amit\METHOD\FUMONISIN\FUMONISIN_02.lcm
Data File	: E:\Amit\BATCH\DATA 2023\JULY 2023\FUMONISIN ON MAIZE\caliberation\SAMPLE 09.lcd

MS Chromatogram

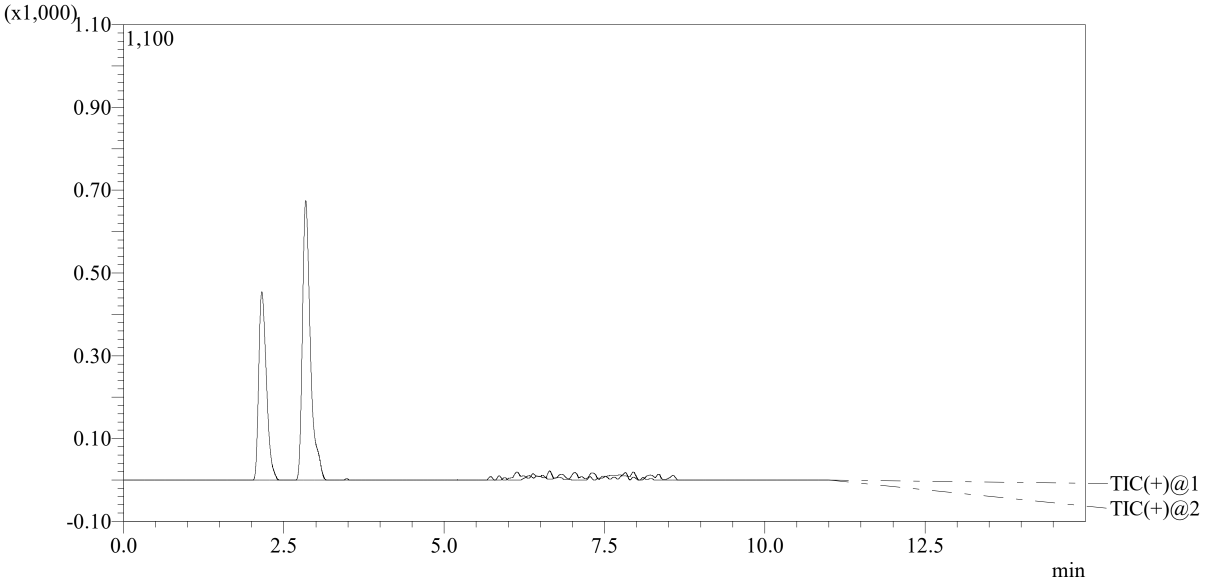

MS Quantitative Graph

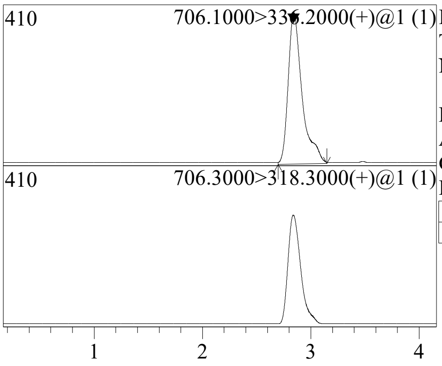

ID#:1 m/z:706.1000>336.2000
Type:Target
Name:FUMANOSIN

Ret.Time:2.841
Area:3538
Conc.:0.007ppm
Event:1

#	m/z	Intensity	Act%
1	706.30>318.30	267	36.63

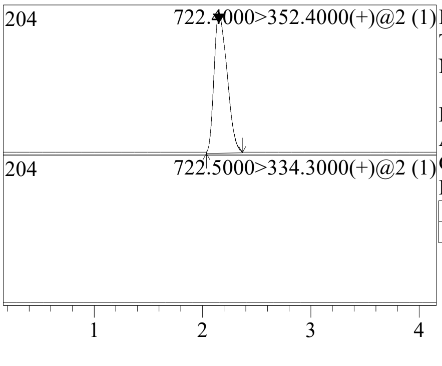

ID#:2 m/z:722.4000>352.4000
Type:Target
Name:FUMONISIN 1

Ret.Time:2.152
Area:1629
Conc.:0.007ppm
Event:2

#	m/z	Intensity	Act%
1	722.50>334.30	218	91.98

MS Quantitative Table

ID#	Name	Ret. Time	m/z	Conc.	Area	Unit
1	FUMANOSIN	2.841	706.10>336.20	0.007	3538	ppm
2	FUMONISIN 1	2.152	722.40>352.40	0.007	1629	ppm
Total					5167	
