# Supplementary material for: Unveiling toxigenic Fusarium species causing maize ear rot: insights into fumonisin production potential
Source: Front Plant Sci. 2025 Mar 21;16:1516644. doi: 10.3389/fpls.2025.1516644 (PMC11968736; doi:10.3389/fpls.2025.1516644)
Supplement: Supplementary file 3 [file SupplementaryFile1.docx]

**Details of supplementary figures**

**
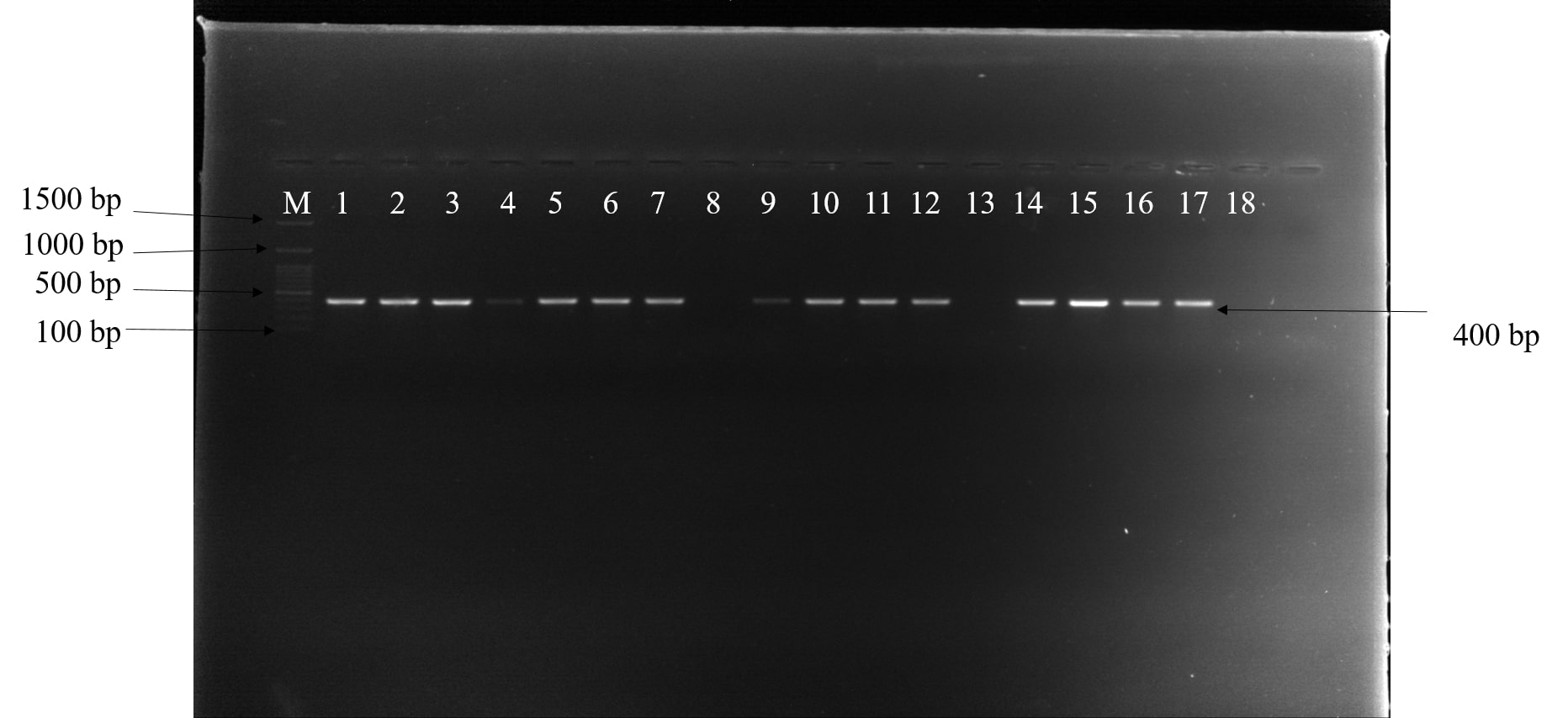
**

**FIGURE S1A**

Amplification of fumonisin producing *F. verticillioides* isolates using VERTF-1/2 primers showing amplification band of 400 bp, Lane M contains- 100 bp ladder; Lane 1-18 contains DNA from *Fusarium* isolates Fus 1 to Fus 18 from different locations

**
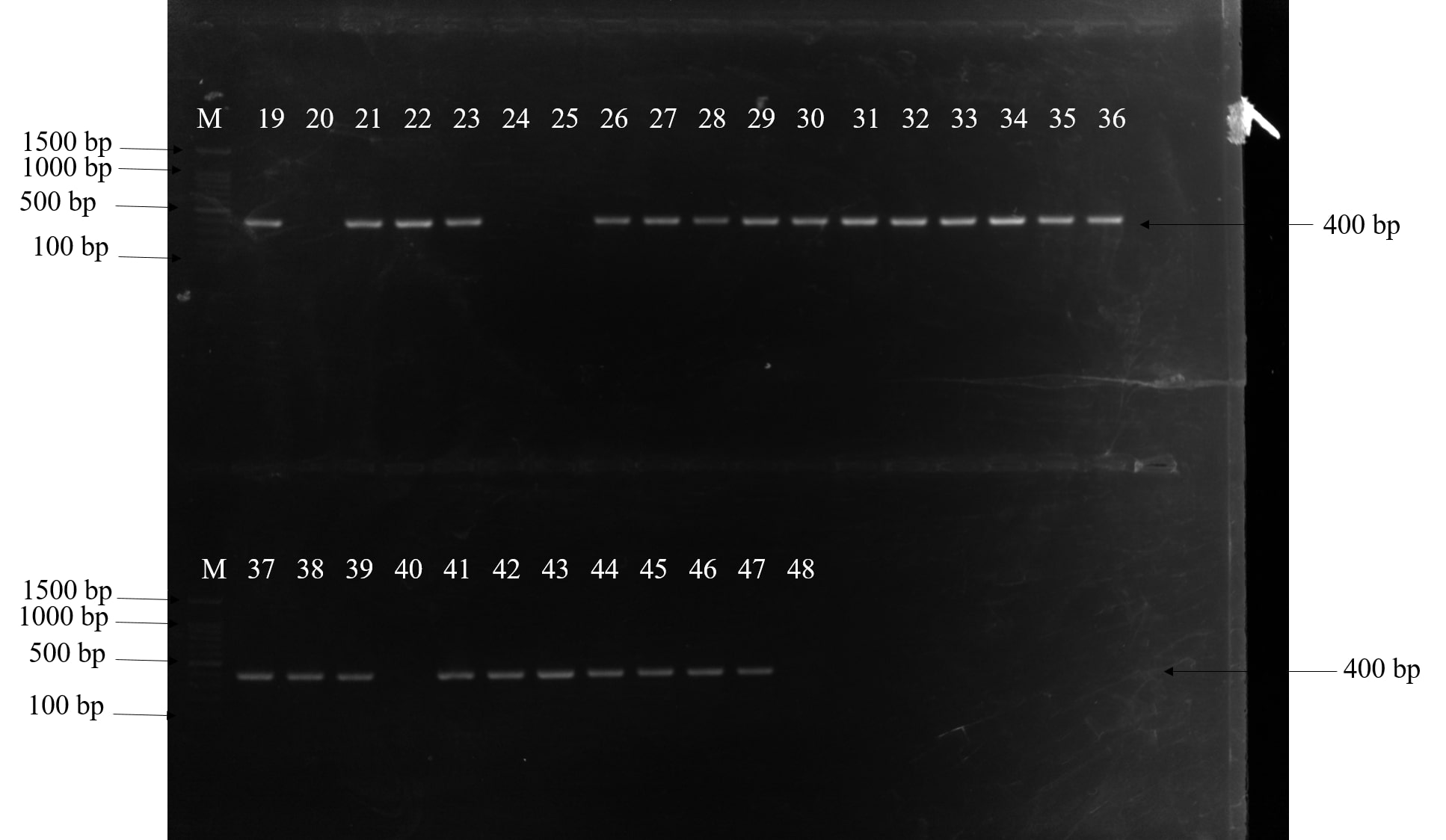
**

**FIGURE S1B**

Amplification of fumonisin producing *F. verticillioides* isolates using VERTF-1/2 primers showing amplification band of 400 bp, Lane M contains- 100 bp ladder; Lane 19-48 contains DNA from *Fusarium* isolates Fus 19 to Fus 48 from different locations

**
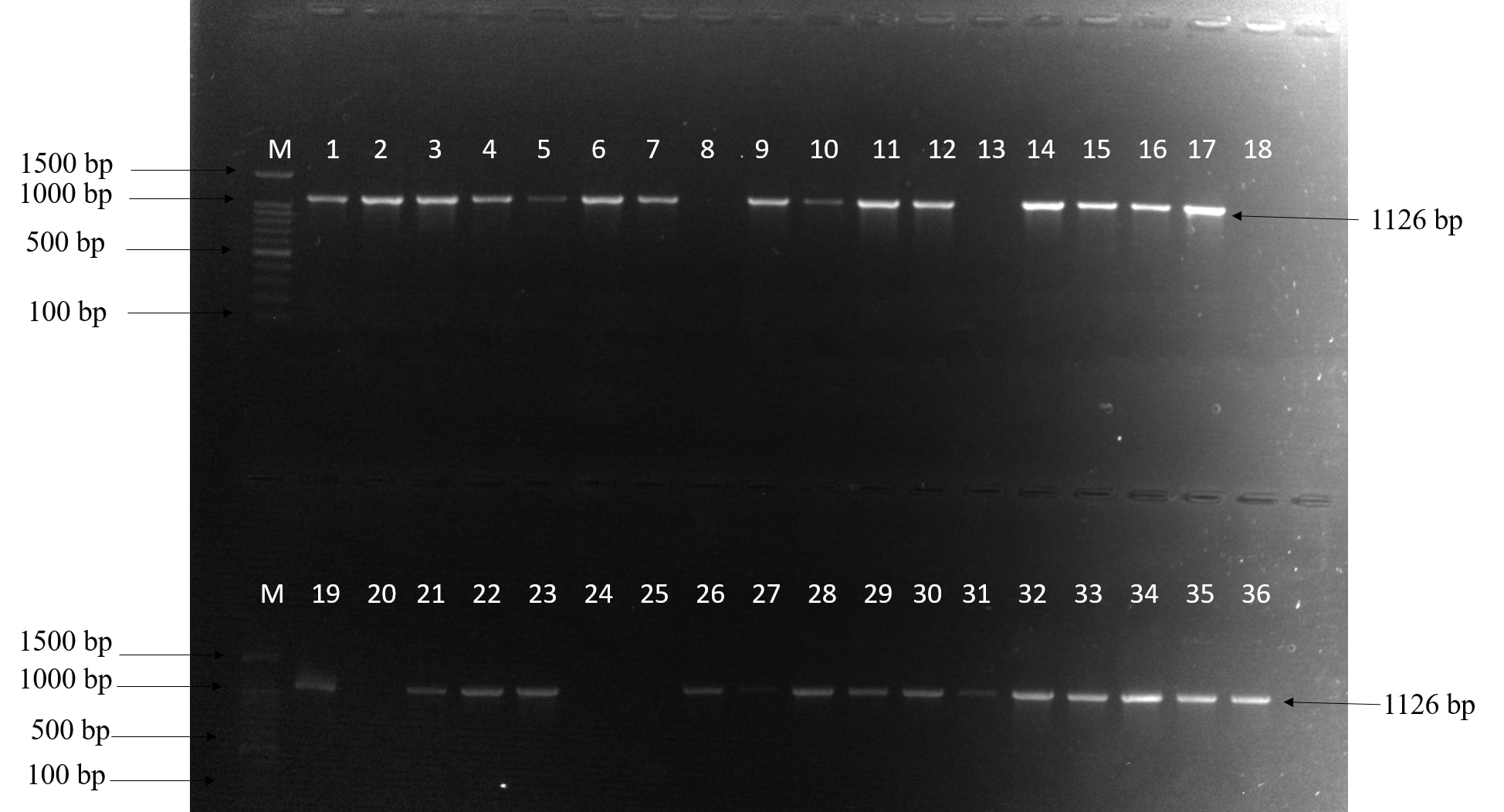
**

**FIGURE S2A**

Amplification of fumonisin producing *Fusarium* isolates using *FUM1* gene specific primers showing amplification band of 1126 bp, Lane M contains- 100 bp ladder; Lane 1-36 contains DNA from *Fusarium* isolates Fus 1 to Fus 36 from different locations

**
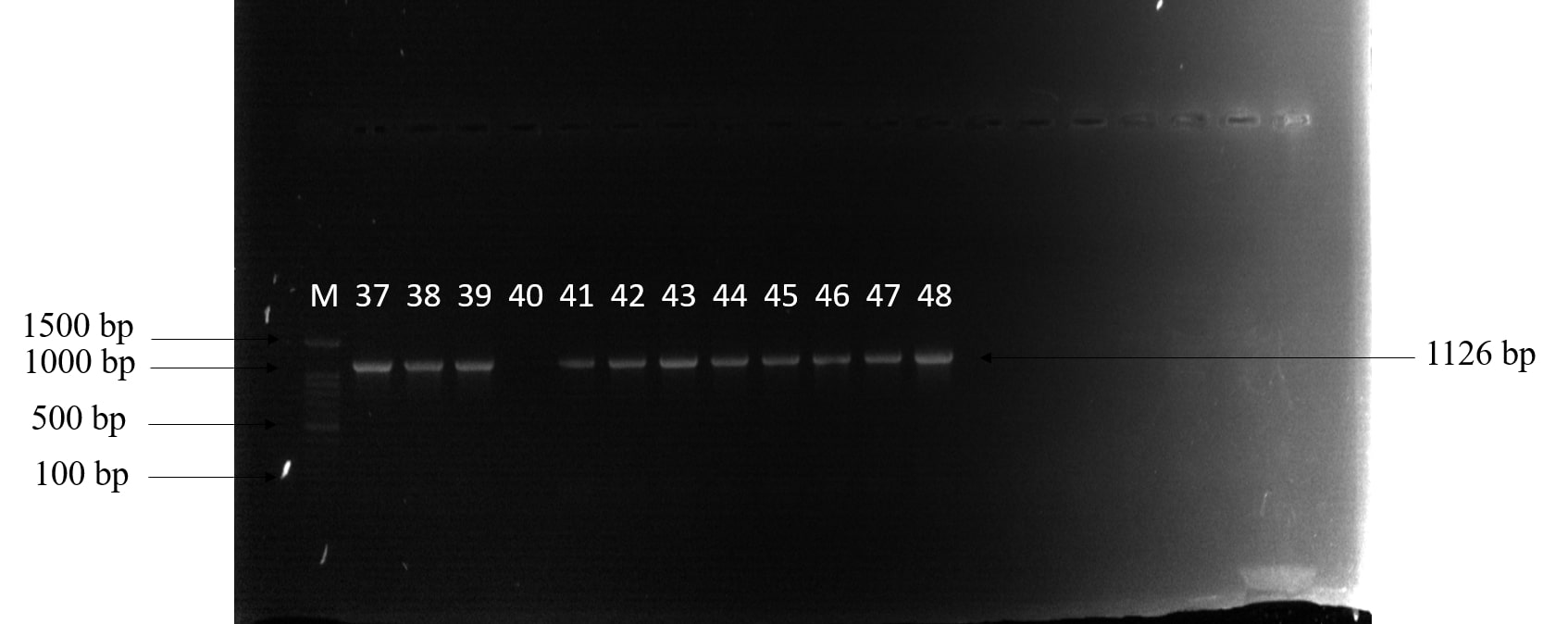
**

**FIGURE S2B**

Amplification of fumonisin producing *Fusarium* isolates using *FUM1* gene specific primers showing amplification band of 1126 bp, Lane M contains- 100 bp ladder; Lane 37-48 contains DNA from *Fusarium* isolates Fus 37 to Fus 48 from different locations

**
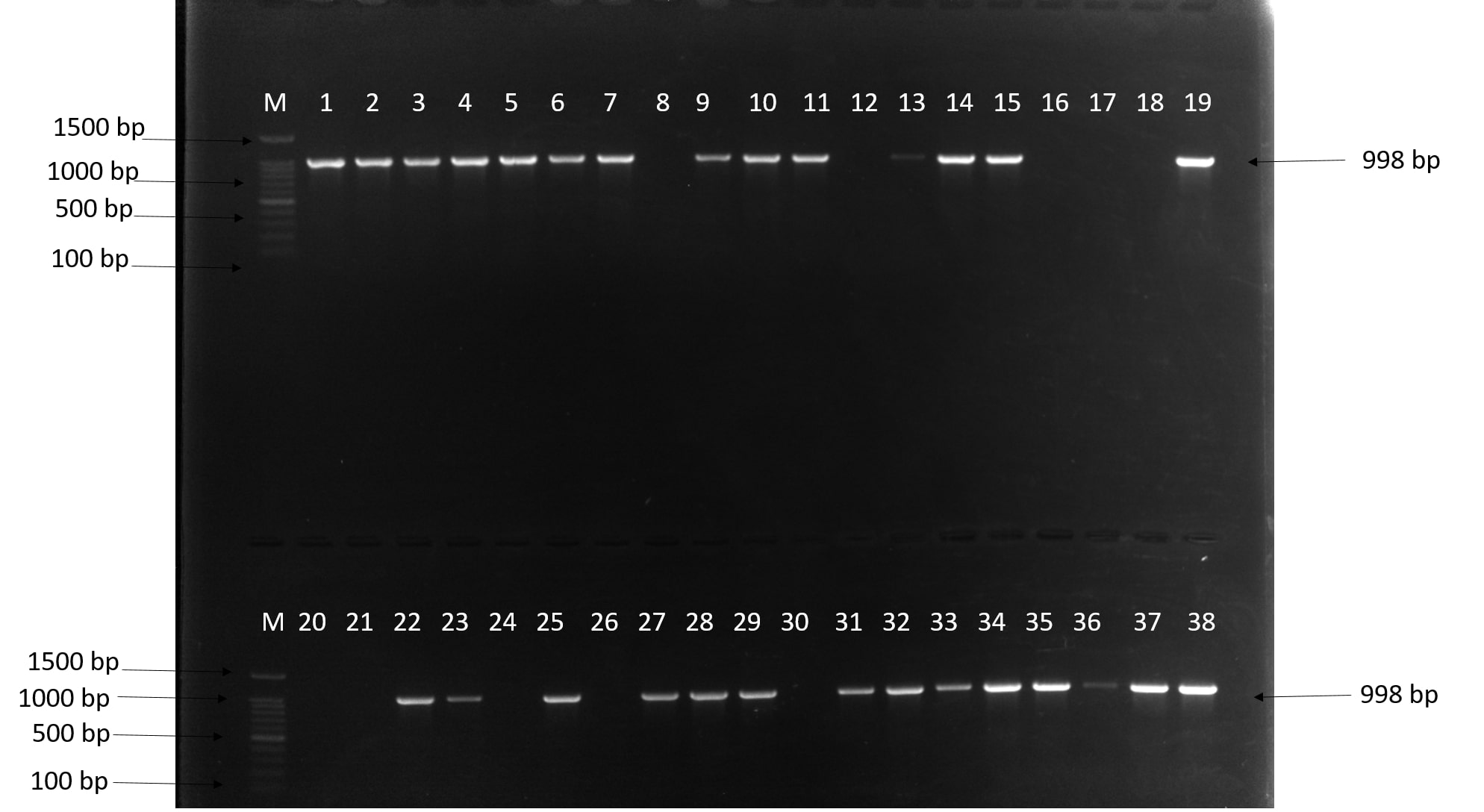
**

**FIGURE S3A**

Amplification of fumonisin producing *Fusarium* isolates using *FUM13* gene specific primers showing amplification band of 998 bp, Lane M contains- 100 bp ladder; Lane 1-38 contains DNA from *Fusarium* isolates Fus 1 to Fus 38 from different locations

**
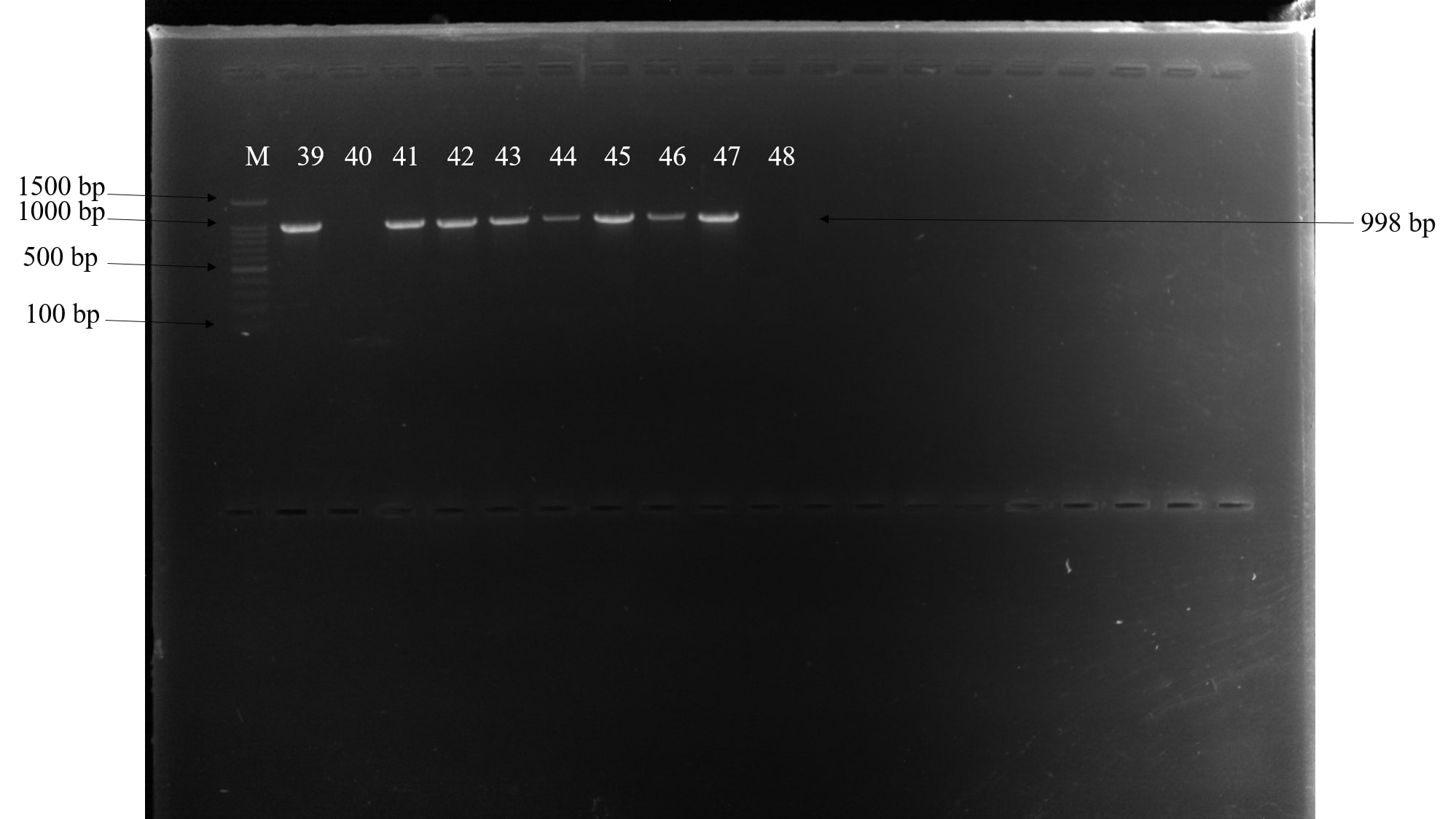
**

**FIGURE S3B**

Amplification of fumonisin producing *Fusarium* isolates using *FUM13* gene specific primers showing amplification band of 998 bp, Lane M contains- 100 bp ladder; Lane 39-48 contains DNA from *Fusarium* isolates Fus 39 to Fus 48 from different locations


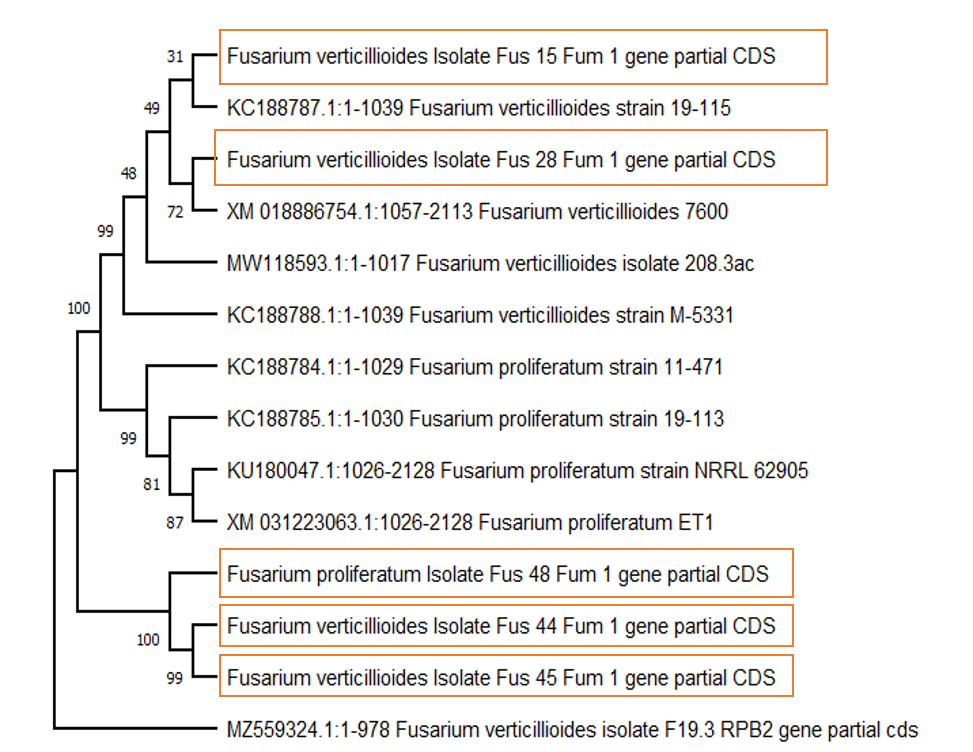


**Figure S4**

**Phylogenetic analysis of fumonisin biosynthesis gene *Fum1* from selected *Fusarium* isolates from this study**

**
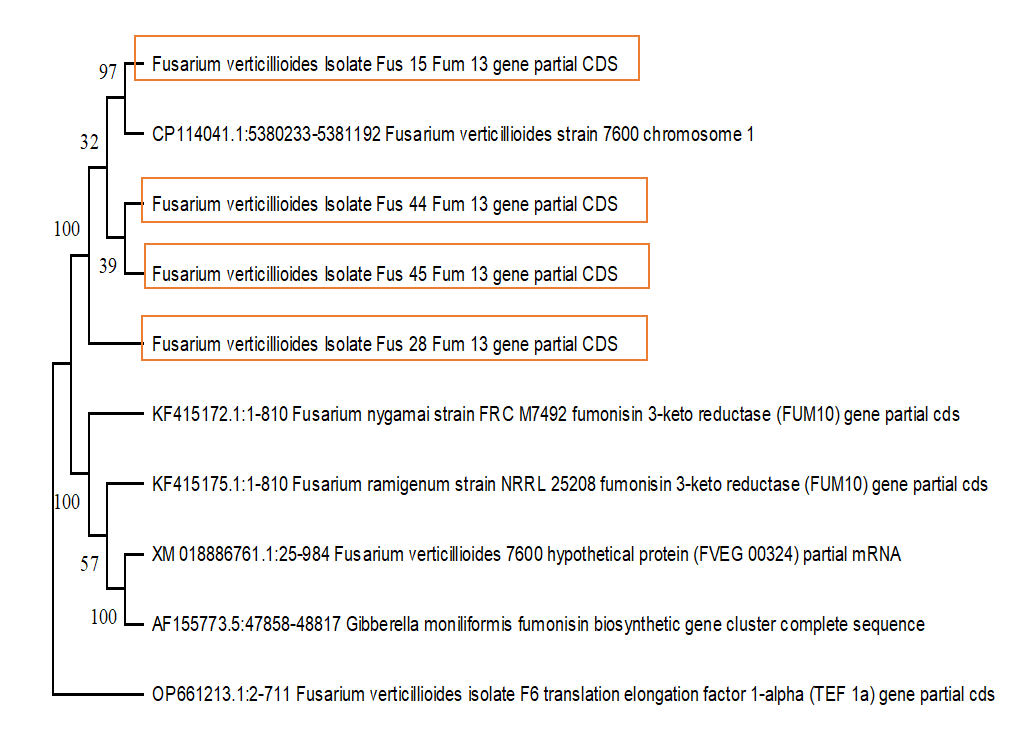
**

**Figure S5**

**Phylogenetic analysis of fumonisin biosynthesis gene *Fum13* from selected *Fusarium* isolates from this study**

**c)**

**Figure S6**

**Fumonisin production in maize grains artificially inoculated** **with *Fusarium verticillioides* isolate (Fus 15) and *Fusarium proliferatum* isolate (Fus 48) a) FB_1_ production (Fus 15)** **b) FB_1_ production (Fus 48) c) FB_2_ production (Fus 15) d) FB_2_ production (Fus 48)**
